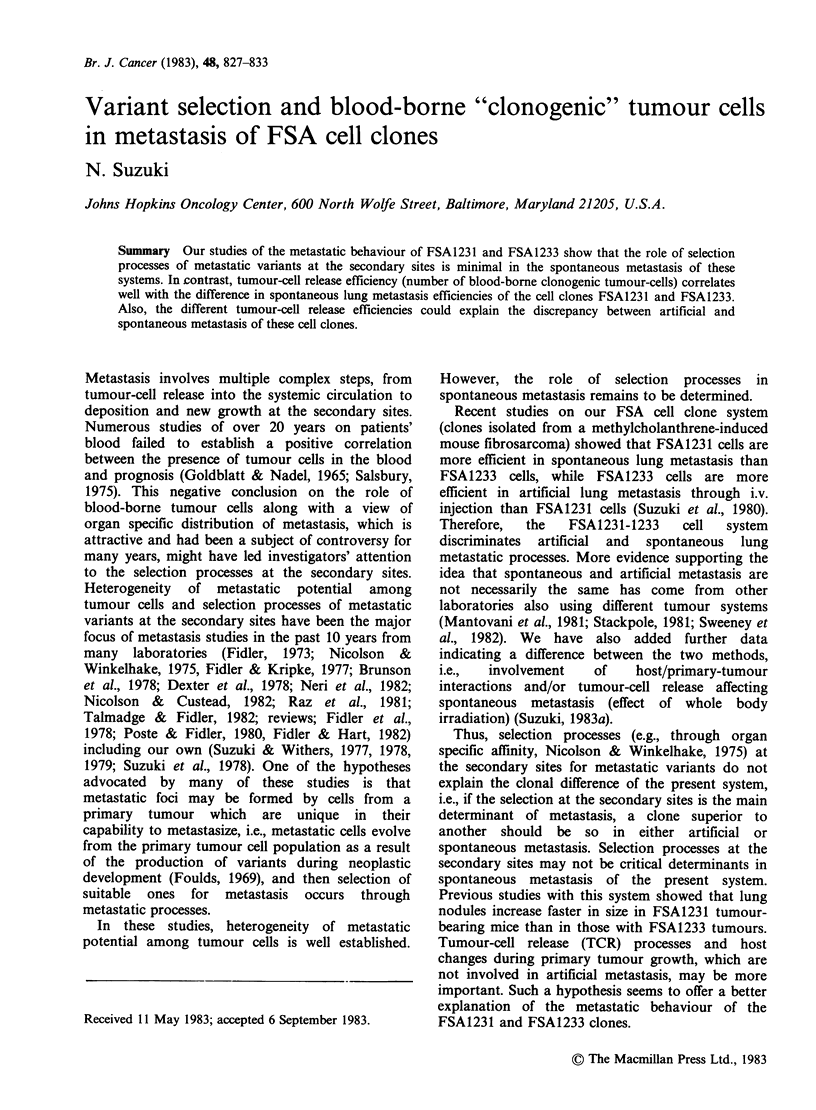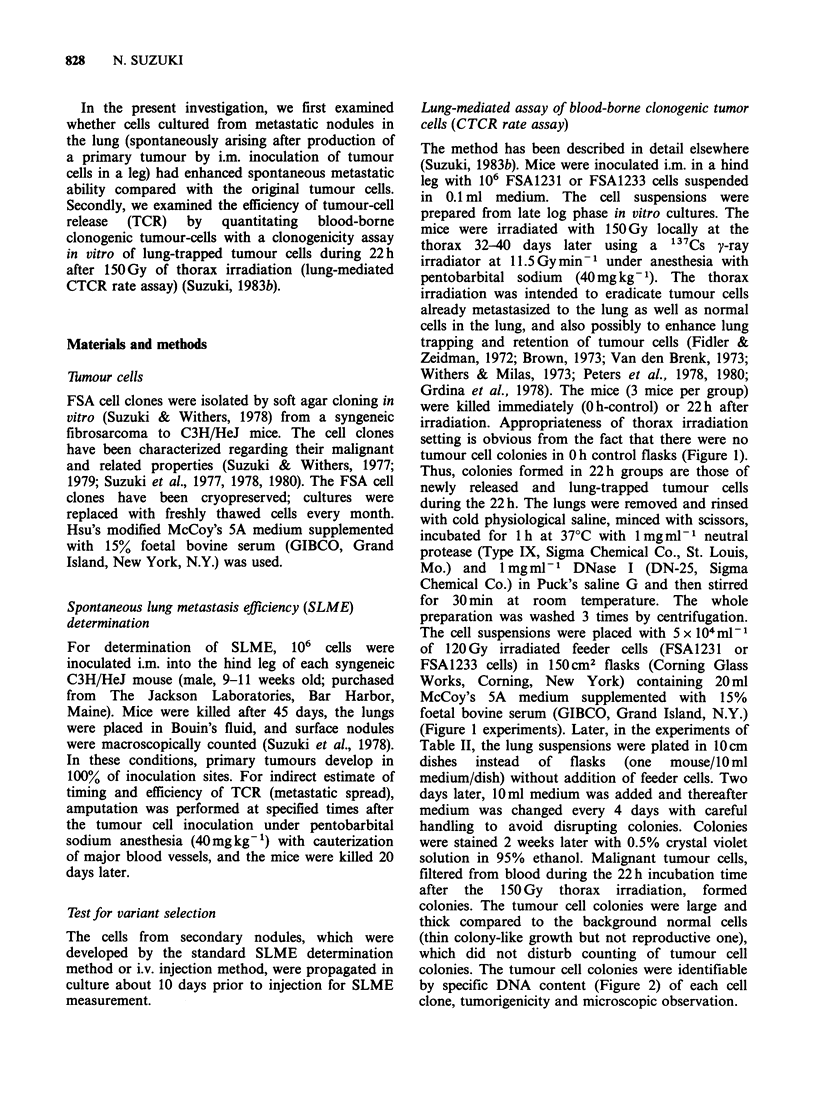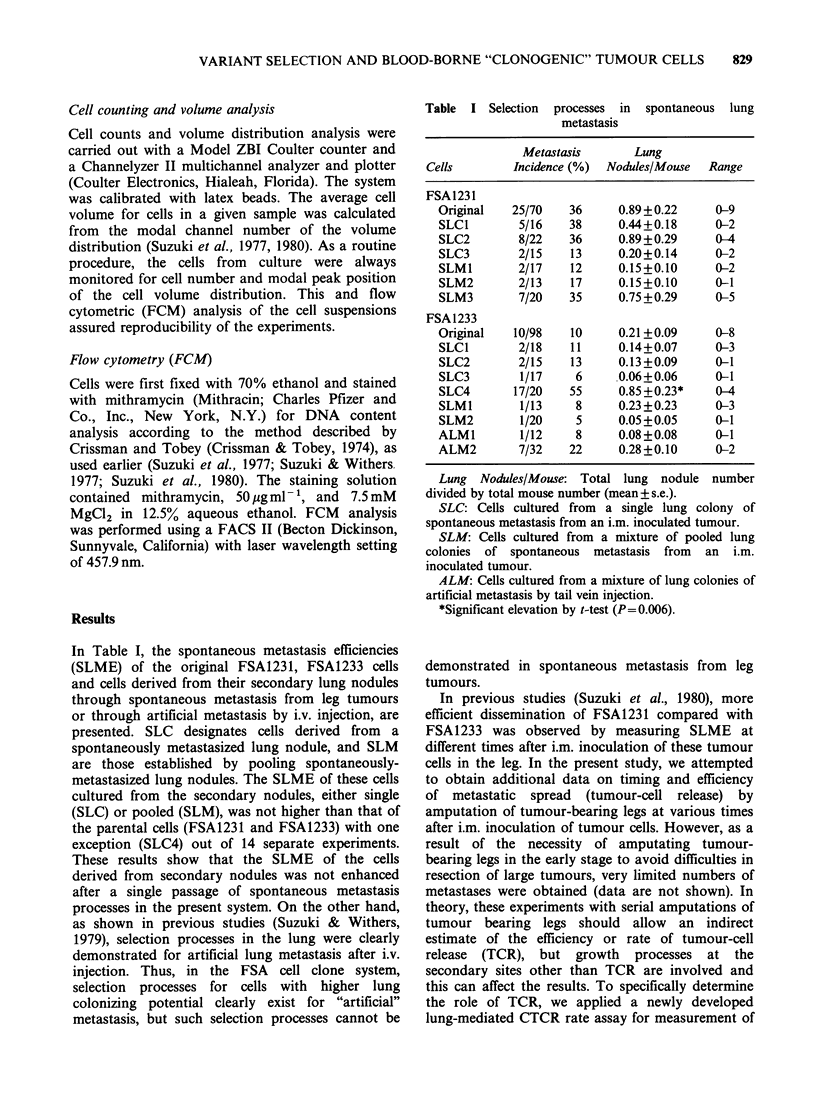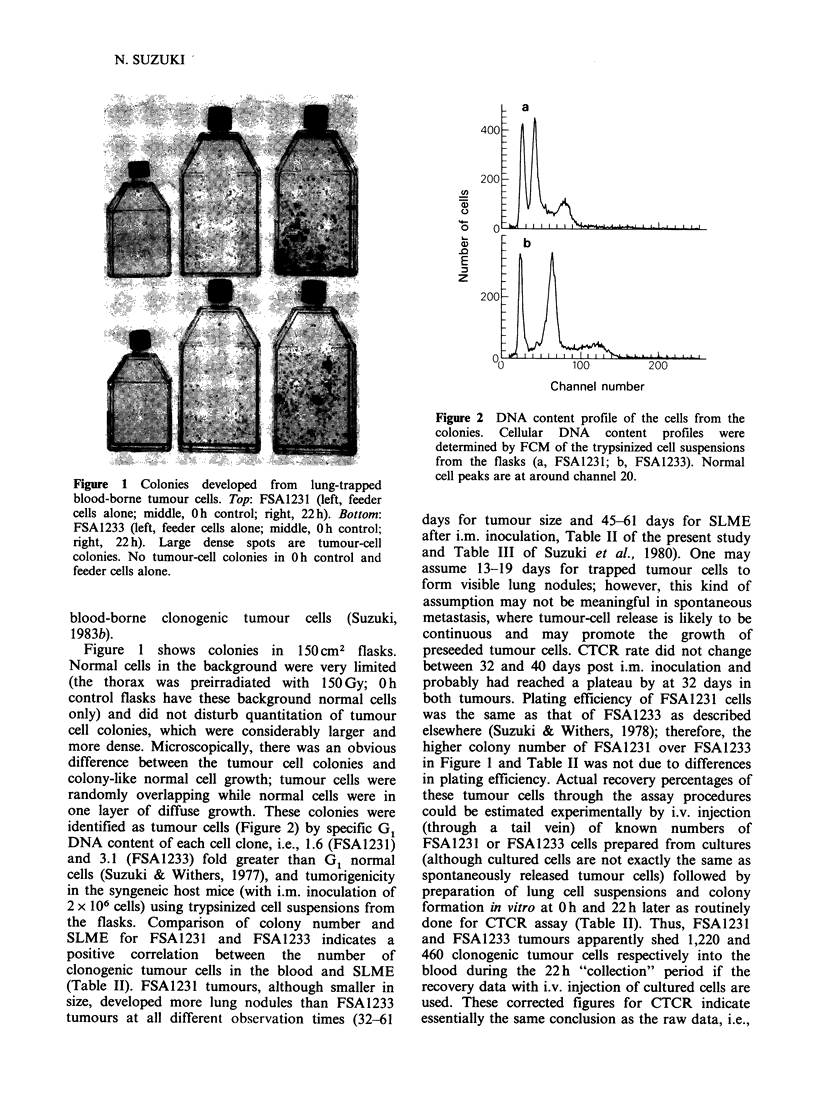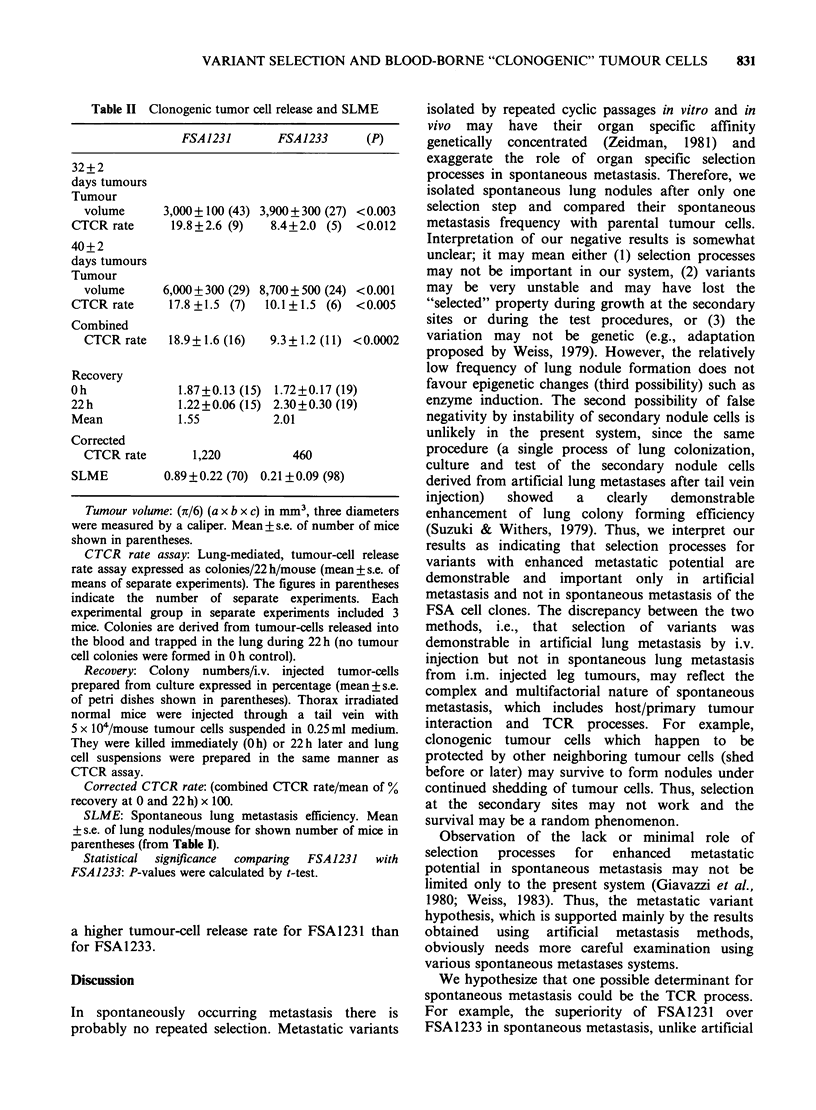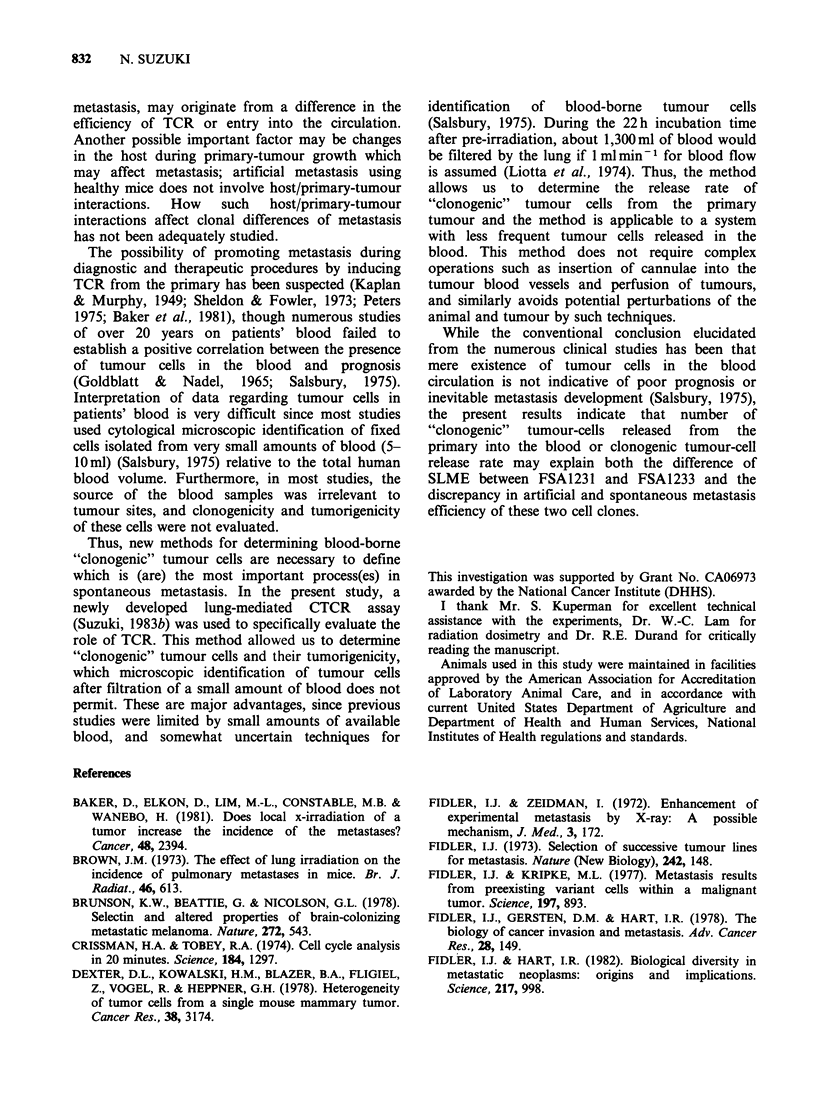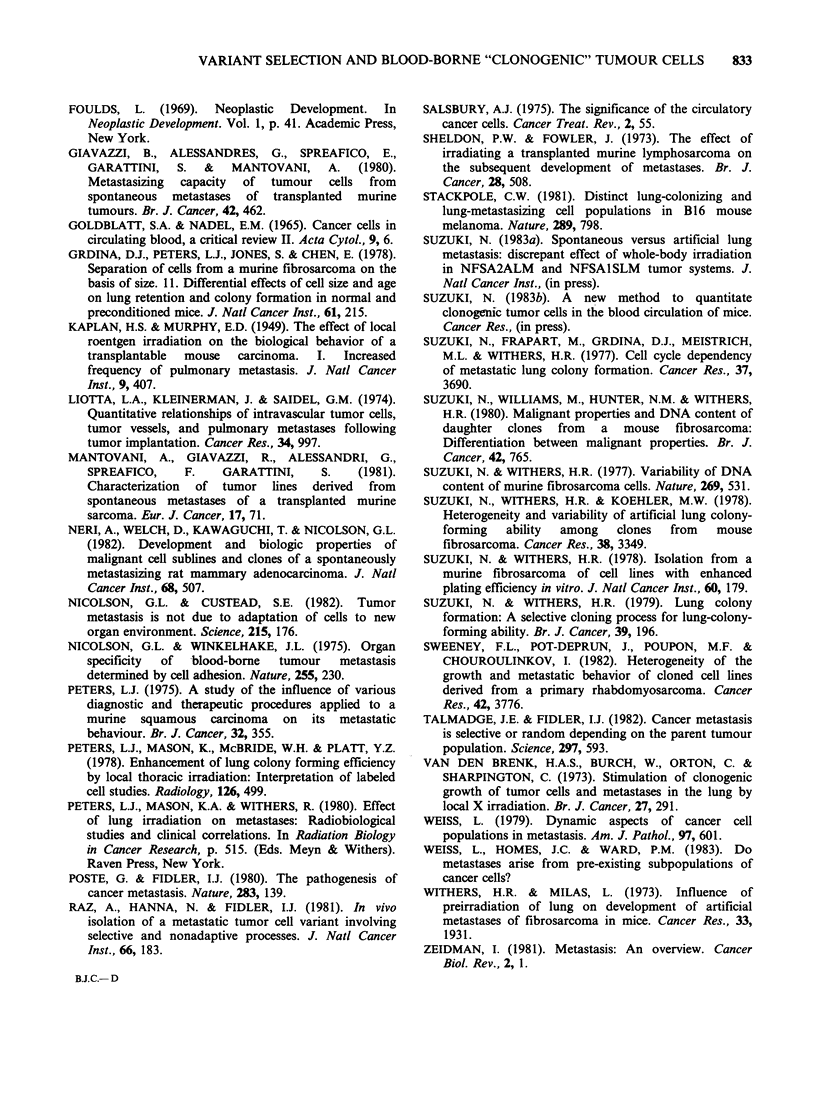# Variant selection and blood-borne "clonogenic" tumour cells in metastasis of FSA cell clones.

**DOI:** 10.1038/bjc.1983.273

**Published:** 1983-12

**Authors:** N. Suzuki

## Abstract

**Images:**


*Br. J. Cancer* (1983), **48**, 827–833

### Variant selection and blood-borne "clonogenic" tumour cells in metastasis of FSA cell clones

N. Suzuki

*Johns Hopkins Oncology Center, 600 North Wolfe Street, Baltimore, Maryland 21205, U.S.A.*

**Summary** Our studies of the metastatic behaviour of FSA1231 and FSA1233 show that the role of selection processes of metastatic variants at the secondary sites is minimal in the spontaneous metastasis of these systems. In contrast, tumour-cell release efficiency (number of blood-borne clonogenic tumour-cells) correlates well with the difference in spontaneous lung metastasis efficiencies of the cell clones FSA1231 and FSA1233. Also, the different tumour-cell release efficiencies could explain the discrepancy between artificial and spontaneous metastasis of these cell clones.

Metastasis involves multiple complex steps, from tumour-cell release into the systemic circulation to deposition and new growth at the secondary sites. Numerous studies of over 20 years on patients' blood failed to establish a positive correlation between the presence of tumour cells in the blood and prognosis (Goldblatt & Nadel, 1965; Salsbury, 1975). This negative conclusion on the role of blood-borne tumour cells along with a view of organ specific distribution of metastasis, which is attractive and had been a subject of controversy for many years, might have led investigators' attention to the selection processes at the secondary sites. Heterogeneity of metastatic potential among tumour cells and selection processes of metastatic variants at the secondary sites have been the major focus of metastasis studies in the past 10 years from many laboratories (Fidler, 1973; Nicolson & Winkelhake, 1975, Fidler & Kripke, 1977; Brunson *et al.*, 1978; Dexter *et al.*, 1978; Neri *et al.*, 1982; Nicolson & Custead, 1982; Raz *et al.*, 1981; Talmadge & Fidler, 1982; reviews; Fidler *et al.*, 1978; Poste & Fidler, 1980, Fidler & Hart, 1982) including our own (Suzuki & Withers, 1977, 1978, 1979; Suzuki *et al.*, 1978). One of the hypotheses advocated by many of these studies is that metastatic foci may be formed by cells from a primary tumour which are unique in their capability to metastasize, i.e., metastatic cells evolve from the primary tumour cell population as a result of the production of variants during neoplastic development (Foulds, 1969), and then selection of suitable ones for metastasis occurs through metastatic processes.

In these studies, heterogeneity of metastatic potential among tumour cells is well established. However, the role of selection processes in spontaneous metastasis remains to be determined.

Recent studies on our FSA cell clone system (clones isolated from a methylcholanthrene-induced mouse fibrosarcoma) showed that FSA1231 cells are more efficient in spontaneous lung metastasis than FSA1233 cells, while FSA1233 cells are more efficient in artificial lung metastasis through i.v. injection than FSA1231 cells (Suzuki *et al.*, 1980). Therefore, the FSA1231-1233 cell system discriminates artificial and spontaneous lung metastatic processes. More evidence supporting the idea that spontaneous and artificial metastasis are not necessarily the same has come from other laboratories also using different tumour systems (Mantovani *et al.*, 1981; Stackpole, 1981; Sweeney *et al.*, 1982). We have also added further data indicating a difference between the two methods, i.e., involvement of host/primary-tumour interactions and/or tumour-cell release affecting spontaneous metastasis (effect of whole body irradiation) (Suzuki, 1983*a*).

Thus, selection processes (e.g., through organ specific affinity, Nicolson & Winkelhake, 1975) at the secondary sites for metastatic variants do not explain the clonal difference of the present system, i.e., if the selection at the secondary sites is the main determinant of metastasis, a clone superior to another should be so in either artificial or spontaneous metastasis. Selection processes at the secondary sites may not be critical determinants in spontaneous metastasis of the present system. Previous studies with this system showed that lung nodules increase faster in size in FSA1231 tumour-bearing mice than in those with FSA1233 tumours. Tumour-cell release (TCR) processes and host changes during primary tumour growth, which are not involved in artificial metastasis, may be more important. Such a hypothesis seems to offer a better explanation of the metastatic behaviour of the FSA1231 and FSA1233 clones.

Received 11 May 1983; accepted 6 September 1983.

© The Macmillan Press Ltd., 1983

In the present investigation, we first examined whether cells cultured from metastatic nodules in the lung (spontaneously arising after production of a primary tumour by i.m. inoculation of tumour cells in a leg) had enhanced spontaneous metastatic ability compared with the original tumour cells. Secondly, we examined the efficiency of tumour-cell release (TCR) by quantitating blood-borne clonogenic tumour-cells with a clonogenicity assay *in vitro* of lung-trapped tumour cells during 22 h after 150 Gy of thorax irradiation (lung-mediated CTCR rate assay) (Suzuki, 1983*b*).

### Materials and methods

### *Tumour cells*

FSA cell clones were isolated by soft agar cloning *in vitro* (Suzuki & Withers, 1978) from a syngeneic fibrosarcoma to C3H/HeJ mice. The cell clones have been characterized regarding their malignant and related properties (Suzuki & Withers, 1977; 1979; Suzuki *et al.*, 1977, 1978, 1980). The FSA cell clones have been cryopreserved; cultures were replaced with freshly thawed cells every month. Hsu's modified McCoy's 5A medium supplemented with 15% foetal bovine serum (GIBCO, Grand Island, New York, N.Y.) was used.

### *Spontaneous lung metastasis efficiency (SLME) determination*

For determination of SLME, $10^6$ cells were inoculated i.m. into the hind leg of each syngeneic C3H/HeJ mouse (male, 9–11 weeks old; purchased from The Jackson Laboratories, Bar Harbor, Maine). Mice were killed after 45 days, the lungs were placed in Bouin's fluid, and surface nodules were macroscopically counted (Suzuki *et al.*, 1978). In these conditions, primary tumours develop in 100% of inoculation sites. For indirect estimate of timing and efficiency of TCR (metastatic spread), amputation was performed at specified times after the tumour cell inoculation under pentobarbital sodium anesthesia ($40\,mg\,kg^{-1}$) with cauterization of major blood vessels, and the mice were killed 20 days later.

### *Test for variant selection*

The cells from secondary nodules, which were developed by the standard SLME determination method or i.v. injection method, were propagated in culture about 10 days prior to injection for SLME measurement.

### *Lung-mediated assay of blood-borne clonogenic tumor cells (CTCR rate assay)*

The method has been described in detail elsewhere (Suzuki, 1983*b*). Mice were inoculated i.m. in a hind leg with $10^6$ FSA1231 or FSA1233 cells suspended in 0.1 ml medium. The cell suspensions were prepared from late log phase *in vitro* cultures. The mice were irradiated with 150 Gy locally at the thorax 32–40 days later using a $^{137}Cs$ $\gamma$-ray irradiator at $11.5\,Gy\,min^{-1}$ under anesthesia with pentobarbital sodium ($40\,mg\,kg^{-1}$). The thorax irradiation was intended to eradicate tumour cells already metastasized to the lung as well as normal cells in the lung, and also possibly to enhance lung trapping and retention of tumour cells (Fidler & Zeidman, 1972; Brown, 1973; Van den Brenk, 1973; Withers & Milas, 1973; Peters *et al.*, 1978, 1980; Grdina *et al.*, 1978). The mice (3 mice per group) were killed immediately (0 h-control) or 22 h after irradiation. Appropriateness of thorax irradiation setting is obvious from the fact that there were no tumour cell colonies in 0 h control flasks (Figure 1). Thus, colonies formed in 22 h groups are those of newly released and lung-trapped tumour cells during the 22 h. The lungs were removed and rinsed with cold physiological saline, minced with scissors, incubated for 1 h at 37°C with $1\,mg\,ml^{-1}$ neutral protease (Type IX, Sigma Chemical Co., St. Louis, Mo.) and $1\,mg\,ml^{-1}$ DNase I (DN-25, Sigma Chemical Co.) in Puck's saline G and then stirred for 30 min at room temperature. The whole preparation was washed 3 times by centrifugation. The cell suspensions were placed with $5\times10^4\,ml^{-1}$ of 120 Gy irradiated feeder cells (FSA1231 or FSA1233 cells) in $150\,cm^2$ flasks (Corning Glass Works, Corning, New York) containing 20 ml McCoy's 5A medium supplemented with 15% foetal bovine serum (GIBCO, Grand Island, N.Y.) (Figure 1 experiments). Later, in the experiments of Table II, the lung suspensions were plated in 10 cm dishes instead of flasks (one mouse/10 ml medium/dish) without addition of feeder cells. Two days later, 10 ml medium was added and thereafter medium was changed every 4 days with careful handling to avoid disrupting colonies. Colonies were stained 2 weeks later with 0.5% crystal violet solution in 95% ethanol. Malignant tumour cells, filtered from blood during the 22 h incubation time after the 150 Gy thorax irradiation, formed colonies. The tumour cell colonies were large and thick compared to the background normal cells (thin colony-like growth but not reproductive one), which did not disturb counting of tumour cell colonies. The tumour cell colonies were identifiable by specific DNA content (Figure 2) of each cell clone, tumorigenicity and microscopic observation.

### *Cell counting and volume analysis*

Cell counts and volume distribution analysis were carried out with a Model ZBI Coulter counter and a Channelyzer II multichannel analyzer and plotter (Coulter Electronics, Hialeah, Florida). The system was calibrated with latex beads. The average cell volume for cells in a given sample was calculated from the modal channel number of the volume distribution (Suzuki *et al.*, 1977, 1980). As a routine procedure, the cells from culture were always monitored for cell number and modal peak position of the cell volume distribution. This and flow cytometric (FCM) analysis of the cell suspensions assured reproducibility of the experiments.

### *Flow cytometry (FCM)*

Cells were first fixed with 70% ethanol and stained with mithramycin (Mithracin; Charles Pfizer and Co., Inc., New York, N.Y.) for DNA content analysis according to the method described by Crissman and Tobey (Crissman & Tobey, 1974), as used earlier (Suzuki *et al.*, 1977; Suzuki & Withers 1977; Suzuki *et al.*, 1980). The staining solution contained mithramycin, $50\,\mu g\,ml^{-1}$, and 7.5 mM $MgCl_2$ in 12.5% aqueous ethanol. FCM analysis was performed using a FACS II (Becton Dickinson, Sunnyvale, California) with laser wavelength setting of 457.9 nm.

### Results

In Table I, the spontaneous metastasis efficiencies (SLME) of the original FSA1231, FSA1233 cells and cells derived from their secondary lung nodules through spontaneous metastasis from leg tumours or through artificial metastasis by i.v. injection, are presented. SLC designates cells derived from a spontaneously metastasized lung nodule, and SLM are those established by pooling spontaneously-metastasized lung nodules. The SLME of these cells cultured from the secondary nodules, either single (SLC) or pooled (SLM), was not higher than that of the parental cells (FSA1231 and FSA1233) with one exception (SLC4) out of 14 separate experiments. These results show that the SLME of the cells derived from secondary nodules was not enhanced after a single passage of spontaneous metastasis processes in the present system. On the other hand, as shown in previous studies (Suzuki & Withers, 1979), selection processes in the lung were clearly demonstrated for artificial lung metastasis after i.v. injection. Thus, in the FSA cell clone system, selection processes for cells with higher lung colonizing potential clearly exist for "artificial" metastasis, but such selection processes cannot be demonstrated in spontaneous metastasis from leg tumours.

**Table I** Selection processes in spontaneous lung metastasis

| *Cells* | *Metastasis Incidence* | (%) | *Lung Nodules/Mouse* | *Range* |
|---|---|---|---|---|
| FSA1231 | | | | |
| Original | 25/70 | 36 | 0.89±0.22 | 0–9 |
| SLC1 | 5/16 | 38 | 0.44±0.18 | 0–2 |
| SLC2 | 8/22 | 36 | 0.89±0.29 | 0–4 |
| SLC3 | 2/15 | 13 | 0.20±0.14 | 0–2 |
| SLM1 | 2/17 | 12 | 0.15±0.10 | 0–2 |
| SLM2 | 2/13 | 17 | 0.15±0.10 | 0–1 |
| SLM3 | 7/20 | 35 | 0.75±0.29 | 0–5 |
| FSA1233 | | | | |
| Original | 10/98 | 10 | 0.21±0.09 | 0–8 |
| SLC1 | 2/18 | 11 | 0.14±0.07 | 0–3 |
| SLC2 | 2/15 | 13 | 0.13±0.09 | 0–1 |
| SLC3 | 1/17 | 6 | 0.06±0.06 | 0–1 |
| SLC4 | 17/20 | 55 | 0.85±0.23* | 0–4 |
| SLM1 | 1/13 | 8 | 0.23±0.23 | 0–3 |
| SLM2 | 1/20 | 5 | 0.05±0.05 | 0–1 |
| ALM1 | 1/12 | 8 | 0.08±0.08 | 0–1 |
| ALM2 | 7/32 | 22 | 0.28±0.10 | 0–2 |

*Lung Nodules/Mouse*: Total lung nodule number divided by total mouse number (mean±s.e.).

*SLC*: Cells cultured from a single lung colony of spontaneous metastasis from an i.m. inoculated tumour.

*SLM*: Cells cultured from a mixture of pooled lung colonies of spontaneous metastasis from an i.m. inoculated tumour.

*ALM*: Cells cultured from a mixture of lung colonies of artificial metastasis by tail vein injection.

*Significant elevation by *t*-test ($P=0.006$).

In previous studies (Suzuki *et al.*, 1980), more efficient dissemination of FSA1231 compared with FSA1233 was observed by measuring SLME at different times after i.m. inoculation of these tumour cells in the leg. In the present study, we attempted to obtain additional data on timing and efficiency of metastatic spread (tumour-cell release) by amputation of tumour-bearing legs at various times after i.m. inoculation of tumour cells. However, as a result of the necessity of amputating tumour-bearing legs in the early stage to avoid difficulties in resection of large tumours, very limited numbers of metastases were obtained (data are not shown). In theory, these experiments with serial amputations of tumour bearing legs should allow an indirect estimate of the efficiency or rate of tumour-cell release (TCR), but growth processes at the secondary sites other than TCR are involved and this can affect the results. To specifically determine the role of TCR, we applied a newly developed lung-mediated CTCR rate assay for measurement of

**Figure 1** Colonies developed from lung-trapped blood-borne tumour cells. *Top*: FSA1231 (left, feeder cells alone; middle, 0 h control; right, 22 h). *Bottom*: FSA1233 (left, feeder cells alone; middle, 0 h control; right, 22 h). Large dense spots are tumour-cell colonies. No tumour-cell colonies in 0 h control and feeder cells alone.

**Figure 2** DNA content profile of the cells from the colonies. Cellular DNA content profiles were determined by FCM of the trypsinized cell suspensions from the flasks (a, FSA1231; b, FSA1233). Normal cell peaks are at around channel 20.

blood-borne clonogenic tumour cells (Suzuki, 1983*b*).

Figure 1 shows colonies in 150 cm² flasks. Normal cells in the background were very limited (the thorax was preirradiated with 150 Gy; 0 h control flasks have these background normal cells only) and did not disturb quantitation of tumour cell colonies, which were considerably larger and more dense. Microscopically, there was an obvious difference between the tumour cell colonies and colony-like normal cell growth; tumour cells were randomly overlapping while normal cells were in one layer of diffuse growth. These colonies were identified as tumour cells (Figure 2) by specific $G_1$ DNA content of each cell clone, i.e., 1.6 (FSA1231) and 3.1 (FSA1233) fold greater than $G_1$ normal cells (Suzuki & Withers, 1977), and tumorigenicity in the syngeneic host mice (with i.m. inoculation of $2 \times 10^6$ cells) using trypsinized cell suspensions from the flasks. Comparison of colony number and SLME for FSA1231 and FSA1233 indicates a positive correlation between the number of clonogenic tumour cells in the blood and SLME (Table II). FSA1231 tumours, although smaller in size, developed more lung nodules than FSA1233 tumours at all different observation times (32–61 days for tumour size and 45–61 days for SLME after i.m. inoculation, Table II of the present study and Table III of Suzuki *et al.*, 1980). One may assume 13–19 days for trapped tumour cells to form visible lung nodules; however, this kind of assumption may not be meaningful in spontaneous metastasis, where tumour-cell release is likely to be continuous and may promote the growth of preseeded tumour cells. CTCR rate did not change between 32 and 40 days post i.m. inoculation and probably had reached a plateau by at 32 days in both tumours. Plating efficiency of FSA1231 cells was the same as that of FSA1233 as described elsewhere (Suzuki & Withers, 1978); therefore, the higher colony number of FSA1231 over FSA1233 in Figure 1 and Table II was not due to differences in plating efficiency. Actual recovery percentages of these tumour cells through the assay procedures could be estimated experimentally by i.v. injection (through a tail vein) of known numbers of FSA1231 or FSA1233 cells prepared from cultures (although cultured cells are not exactly the same as spontaneously released tumour cells) followed by preparation of lung cell suspensions and colony formation *in vitro* at 0 h and 22 h later as routinely done for CTCR assay (Table II). Thus, FSA1231 and FSA1233 tumours apparently shed 1,220 and 460 clonogenic tumour cells respectively into the blood during the 22 h "collection" period if the recovery data with i.v. injection of cultured cells are used. These corrected figures for CTCR indicate essentially the same conclusion as the raw data, i.e.,

**Table II** Clonogenic tumor cell release and SLME

| | *FSA1231* | *FSA1233* | (*P*) |
|---|---|---|---|
| 32±2 days tumours | | | |
| Tumour volume | 3,000±100 (43) | 3,900±300 (27) | <0.003 |
| CTCR rate | 19.8±2.6 (9) | 8.4±2.0 (5) | <0.012 |
| 40±2 days tumours | | | |
| Tumour volume | 6,000±300 (29) | 8,700±500 (24) | <0.001 |
| CTCR rate | 17.8±1.5 (7) | 10.1±1.5 (6) | <0.005 |
| Combined CTCR rate | 18.9±1.6 (16) | 9.3±1.2 (11) | <0.0002 |
| Recovery | | | |
| 0 h | 1.87±0.13 (15) | 1.72±0.17 (19) | |
| 22 h | 1.22±0.06 (15) | 2.30±0.30 (19) | |
| Mean | 1.55 | 2.01 | |
| Corrected CTCR rate | 1,220 | 460 | |
| SLME | 0.89±0.22 (70) | 0.21±0.09 (98) | |

*Tumour volume*: $(\pi/6)$ $(a \times b \times c)$ in $mm^3$, three diameters were measured by a caliper. Mean±s.e. of number of mice shown in parentheses.

*CTCR rate assay*: Lung-mediated, tumour-cell release rate assay expressed as colonies/22 h/mouse (mean±s.e. of means of separate experiments). The figures in parentheses indicate the number of separate experiments. Each experimental group in separate experiments included 3 mice. Colonies are derived from tumour-cells released into the blood and trapped in the lung during 22 h (no tumour cell colonies were formed in 0 h control).

*Recovery*: Colony numbers/i.v. injected tumor-cells prepared from culture expressed in percentage (mean±s.e. of petri dishes shown in parentheses). Thorax irradiated normal mice were injected through a tail vein with $5 \times 10^4$/mouse tumour cells suspended in 0.25 ml medium. They were killed immediately (0 h) or 22 h later and lung cell suspensions were prepared in the same manner as CTCR assay.

*Corrected CTCR rate*: (combined CTCR rate/mean of % recovery at 0 and 22 h) × 100.

*SLME*: Spontaneous lung metastasis efficiency. Mean ±s.e. of lung nodules/mouse for shown number of mice in parentheses (from **Table I**).

*Statistical significance comparing FSA1231 with FSA1233*: *P*-values were calculated by *t*-test.

a higher tumour-cell release rate for FSA1231 than for FSA1233.

### Discussion

In spontaneously occurring metastasis there is probably no repeated selection. Metastatic variants isolated by repeated cyclic passages *in vitro* and *in vivo* may have their organ specific affinity genetically concentrated (Zeidman, 1981) and exaggerate the role of organ specific selection processes in spontaneous metastasis. Therefore, we isolated spontaneous lung nodules after only one selection step and compared their spontaneous metastasis frequency with parental tumour cells. Interpretation of our negative results is somewhat unclear; it may mean either (1) selection processes may not be important in our system, (2) variants may be very unstable and may have lost the "selected" property during growth at the secondary sites or during the test procedures, or (3) the variation may not be genetic (e.g., adaptation proposed by Weiss, 1979). However, the relatively low frequency of lung nodule formation does not favour epigenetic changes (third possibility) such as enzyme induction. The second possibility of false negativity by instability of secondary nodule cells is unlikely in the present system, since the same procedure (a single process of lung colonization, culture and test of the secondary nodule cells derived from artificial lung metastases after tail vein injection) showed a clearly demonstrable enhancement of lung colony forming efficiency (Suzuki & Withers, 1979). Thus, we interpret our results as indicating that selection processes for variants with enhanced metastatic potential are demonstrable and important only in artificial metastasis and not in spontaneous metastasis of the FSA cell clones. The discrepancy between the two methods, i.e., that selection of variants was demonstrable in artificial lung metastasis by i.v. injection but not in spontaneous lung metastasis from i.m. injected leg tumours, may reflect the complex and multifactorial nature of spontaneous metastasis, which includes host/primary tumour interaction and TCR processes. For example, clonogenic tumour cells which happen to be protected by other neighboring tumour cells (shed before or later) may survive to form nodules under continued shedding of tumour cells. Thus, selection at the secondary sites may not work and the survival may be a random phenomenon.

Observation of the lack or minimal role of selection processes for enhanced metastatic potential in spontaneous metastasis may not be limited only to the present system (Giavazzi *et al.*, 1980; Weiss, 1983). Thus, the metastatic variant hypothesis, which is supported mainly by the results obtained using artificial metastasis methods, obviously needs more careful examination using various spontaneous metastases systems.

We hypothesize that one possible determinant for spontaneous metastasis could be the TCR process. For example, the superiority of FSA1231 over FSA1233 in spontaneous metastasis, unlike artificial metastasis, may originate from a difference in the efficiency of TCR or entry into the circulation. Another possible important factor may be changes in the host during primary-tumour growth which may affect metastasis; artificial metastasis using healthy mice does not involve host/primary-tumour interactions. How such host/primary-tumour interactions affect clonal differences of metastasis has not been adequately studied.

The possibility of promoting metastasis during diagnostic and therapeutic procedures by inducing TCR from the primary has been suspected (Kaplan & Murphy, 1949; Sheldon & Fowler, 1973; Peters 1975; Baker *et al.*, 1981), though numerous studies of over 20 years on patients' blood failed to establish a positive correlation between the presence of tumour cells in the blood and prognosis (Goldblatt & Nadel, 1965; Salsbury, 1975). Interpretation of data regarding tumour cells in patients' blood is very difficult since most studies used cytological microscopic identification of fixed cells isolated from very small amounts of blood (5–10 ml) (Salsbury, 1975) relative to the total human blood volume. Furthermore, in most studies, the source of the blood samples was irrelevant to tumour sites, and clonogenicity and tumorigenicity of these cells were not evaluated.

Thus, new methods for determining blood-borne "clonogenic" tumour cells are necessary to define which is (are) the most important process(es) in spontaneous metastasis. In the present study, a newly developed lung-mediated CTCR assay (Suzuki, 1983*b*) was used to specifically evaluate the role of TCR. This method allowed us to determine "clonogenic" tumour cells and their tumorigenicity, which microscopic identification of tumour cells after filtration of a small amount of blood does not permit. These are major advantages, since previous studies were limited by small amounts of available blood, and somewhat uncertain techniques for identification of blood-borne tumour cells (Salsbury, 1975). During the 22 h incubation time after pre-irradiation, about 1,300 ml of blood would be filtered by the lung if $1\,ml\,min^{-1}$ for blood flow is assumed (Liotta *et al.*, 1974). Thus, the method allows us to determine the release rate of "clonogenic" tumour cells from the primary tumour and the method is applicable to a system with less frequent tumour cells released in the blood. This method does not require complex operations such as insertion of cannulae into the tumour blood vessels and perfusion of tumours, and similarly avoids potential perturbations of the animal and tumour by such techniques.

While the conventional conclusion elucidated from the numerous clinical studies has been that mere existence of tumour cells in the blood circulation is not indicative of poor prognosis or inevitable metastasis development (Salsbury, 1975), the present results indicate that number of "clonogenic" tumour-cells released from the primary into the blood or clonogenic tumour-cell release rate may explain both the difference of SLME between FSA1231 and FSA1233 and the discrepancy in artificial and spontaneous metastasis efficiency of these two cell clones.

This investigation was supported by Grant No. CA06973 awarded by the National Cancer Institute (DHHS).

I thank Mr. S. Kuperman for excellent technical assistance with the experiments, Dr. W.-C. Lam for radiation dosimetry and Dr. R.E. Durand for critically reading the manuscript.

Animals used in this study were maintained in facilities approved by the American Association for Accreditation of Laboratory Animal Care, and in accordance with current United States Department of Agriculture and Department of Health and Human Services, National Institutes of Health regulations and standards.

### References

BAKER, D., ELKON, D., LIM, M.-L., CONSTABLE, M.B. & WANEBO, H. (1981). Does local x-irradiation of a tumor increase the incidence of the metastases? *Cancer*, **48**, 2394.

BROWN, J.M. (1973). The effect of lung irradiation on the incidence of pulmonary metastases in mice. *Br. J. Radiat.*, **46**, 613.

BRUNSON, K.W., BEATTIE, G. & NICOLSON, G.L. (1978). Selectin and altered properties of brain-colonizing metastatic melanoma. *Nature*, **272**, 543.

CRISSMAN, H.A. & TOBEY, R.A. (1974). Cell cycle analysis in 20 minutes. *Science*, **184**, 1297.

DEXTER, D.L., KOWALSKI, H.M., BLAZER, B.A., FLIGIEL, Z., VOGEL, R. & HEPPNER, G.H. (1978). Heterogeneity of tumor cells from a single mouse mammary tumor. *Cancer Res.*, **38**, 3174.

FIDLER, I.J. & ZEIDMAN, I. (1972). Enhancement of experimental metastasis by X-ray: A possible mechanism, *J. Med.*, **3**, 172.

FIDLER, I.J. (1973). Selection of successive tumour lines for metastasis. *Nature* (New Biology), **242**, 148.

FIDLER, I.J. & KRIPKE, M.L. (1977). Metastasis results from preexisting variant cells within a malignant tumor. *Science*, **197**, 893.

FIDLER, I.J., GERSTEN, D.M. & HART, I.R. (1978). The biology of cancer invasion and metastasis. *Adv. Cancer Res.*, **28**, 149.

FIDLER, I.J. & HART, I.R. (1982). Biological diversity in metastatic neoplasms: origins and implications. *Science*, **217**, 998.

FOULDS, L. (1969). Neoplastic Development. In *Neoplastic Development*. Vol. 1, p. 41. Academic Press, New York.

GIAVAZZI, B., ALESSANDRES, G., SPREAFICO, E., GARATTINI, S. & MANTOVANI, A. (1980). Metastasizing capacity of tumour cells from spontaneous metastases of transplanted murine tumours. *Br. J. Cancer*, **42**, 462.

GOLDBLATT, S.A. & NADEL, E.M. (1965). Cancer cells in circulating blood, a critical review II. *Acta Cytol.*, **9**, 6.

GRDINA, D.J., PETERS, L.J., JONES, S. & CHEN, E. (1978). Separation of cells from a murine fibrosarcoma on the basis of size. 11. Differential effects of cell size and age on lung retention and colony formation in normal and preconditioned mice. *J. Natl Cancer Inst.*, **61**, 215.

KAPLAN, H.S. & MURPHY, E.D. (1949). The effect of local roentgen irradiation on the biological behavior of a transplantable mouse carcinoma. I. Increased frequency of pulmonary metastasis. *J. Natl Cancer Inst.*, **9**, 407.

LIOTTA, L.A., KLEINERMAN, J. & SAIDEL, G.M. (1974). Quantitative relationships of intravascular tumor cells, tumor vessels, and pulmonary metastases following tumor implantation. *Cancer Res.*, **34**, 997.

MANTOVANI, A., GIAVAZZI, R., ALESSANDRI, G., SPREAFICO, F. GARATTINI, S. (1981). Characterization of tumor lines derived from spontaneous metastases of a transplanted murine sarcoma. *Eur. J. Cancer*, **17**, 71.

NERI, A., WELCH, D., KAWAGUCHI, T. & NICOLSON, G.L. (1982). Development and biologic properties of malignant cell sublines and clones of a spontaneously metastasizing rat mammary adenocarcinoma. *J. Natl Cancer Inst.*, **68**, 507.

NICOLSON, G.L. & CUSTEAD, S.E. (1982). Tumor metastasis is not due to adaptation of cells to new organ environment. *Science*, **215**, 176.

NICOLSON, G.L. & WINKELHAKE, J.L. (1975). Organ specificity of blood-borne tumour metastasis determined by cell adhesion. *Nature*, **255**, 230.

PETERS, L.J. (1975). A study of the influence of various diagnostic and therapeutic procedures applied to a murine squamous carcinoma on its metastatic behaviour. *Br. J. Cancer*, **32**, 355.

PETERS, L.J., MASON, K., MCBRIDE, W.H. & PLATT, Y.Z. (1978). Enhancement of lung colony forming efficiency by local thoracic irradiation: Interpretation of labeled cell studies. *Radiology*, **126**, 499.

PETERS, L.J., MASON, K.A. & WITHERS, R. (1980). Effect of lung irradiation on metastases: Radiobiological studies and clinical correlations. In *Radiation Biology in Cancer Research*, p. 515. (Eds. Meyn & Withers). Raven Press, New York.

POSTE, G. & FIDLER, I.J. (1980). The pathogenesis of cancer metastasis. *Nature*, **283**, 139.

RAZ, A., HANNA, N. & FIDLER, I.J. (1981). *In vivo* isolation of a metastatic tumor cell variant involving selective and nonadaptive processes. *J. Natl Cancer Inst.*, **66**, 183.

SALSBURY, A.J. (1975). The significance of the circulatory cancer cells. *Cancer Treat. Rev.*, **2**, 55.

SHELDON, P.W. & FOWLER, J. (1973). The effect of irradiating a transplanted murine lymphosarcoma on the subsequent development of metastases. *Br. J. Cancer*, **28**, 508.

STACKPOLE, C.W. (1981). Distinct lung-colonizing and lung-metastasizing cell populations in B16 mouse melanoma. *Nature*, **289**, 798.

SUZUKI, N. (1983*a*). Spontaneous versus artificial lung metastasis: discrepant effect of whole-body irradiation in NFSA2ALM and NFSA1SLM tumor systems. *J. Natl Cancer Inst.*, (in press).

SUZUKI, N. (1983*b*). A new method to quantitate clonogenic tumor cells in the blood circulation of mice. *Cancer Res.*, (in press).

SUZUKI, N., FRAPART, M., GRDINA, D.J., MEISTRICH, M.L. & WITHERS, H.R. (1977). Cell cycle dependency of metastatic lung colony formation. *Cancer Res.*, **37**, 3690.

SUZUKI, N., WILLIAMS, M., HUNTER, N.M. & WITHERS, H.R. (1980). Malignant properties and DNA content of daughter clones from a mouse fibrosarcoma: Differentiation between malignant properties. *Br. J. Cancer*, **42**, 765.

SUZUKI, N. & WITHERS, H.R. (1977). Variability of DNA content of murine fibrosarcoma cells. *Nature*, **269**, 531.

SUZUKI, N., WITHERS, H.R. & KOEHLER, M.W. (1978). Heterogeneity and variability of artificial lung colony-forming ability among clones from mouse fibrosarcoma. *Cancer Res.*, **38**, 3349.

SUZUKI, N. & WITHERS, H.R. (1978). Isolation from a murine fibrosarcoma of cell lines with enhanced plating efficiency *in vitro*. *J. Natl Cancer Inst.*, **60**, 179.

SUZUKI, N. & WITHERS, H.R. (1979). Lung colony formation: A selective cloning process for lung-colony-forming ability. *Br. J. Cancer*, **39**, 196.

SWEENEY, F.L., POT-DEPRUN, J., POUPON, M.F. & CHOUROULINKOV, I. (1982). Heterogeneity of the growth and metastatic behavior of cloned cell lines derived from a primary rhabdomyosarcoma. *Cancer Res.*, **42**, 3776.

TALMADGE, J.E. & FIDLER, I.J. (1982). Cancer metastasis is selective or random depending on the parent tumour population. *Science*, **297**, 593.

VAN DEN BRENK, H.A.S., BURCH, W., ORTON, C. & SHARPINGTON, C. (1973). Stimulation of clonogenic growth of tumor cells and metastases in the lung by local X irradiation. *Br. J. Cancer*, **27**, 291.

WEISS, L. (1979). Dynamic aspects of cancer cell populations in metastasis. *Am. J. Pathol.*, **97**, 601.

WEISS, L., HOMES, J.C. & WARD, P.M. (1983). Do metastases arise from pre-existing subpopulations of cancer cells?

WITHERS, H.R. & MILAS, L. (1973). Influence of preirradiation of lung on development of artificial metastases of fibrosarcoma in mice. *Cancer Res.*, **33**, 1931.

ZEIDMAN, I. (1981). Metastasis: An overview. *Cancer Biol. Rev.*, **2**, 1.